# Conditioned Medium from Canine Amniotic Membrane-Derived Mesenchymal Stem Cells Improved Dog Sperm Post-Thaw Quality-Related Parameters

**DOI:** 10.3390/ani10101899

**Published:** 2020-10-16

**Authors:** Feriel Yasmine Mahiddine, Jin Wook Kim, Ahmad Yar Qamar, Jeong Chan Ra, Soo Hyun Kim, Eun Joong Jung, Min Jung Kim

**Affiliations:** 1 Department of Theriogenology and Biotechnologies, College of Veterinary Medicine, Seoul National University, Seoul 08826, Korea; yasmini19@snu.ac.kr (F.Y.M.); vet_chris@snu.ac.kr (J.W.K.); 2 Laboratory of Theriogenology, College of Veterinary Medicine, Chungnam National University, Daejeon 34134, Korea; ahmad.qamar@uvas.edu.pk; 3 Department of Clinical Sciences, College of Veterinary and Animal Sciences, Jhang 35200, Pakistan, Sub-Campus University of Veterinary and Animal Sciences, Lahore 54000, Pakistan; 4 Cell Physiology Research Center, Naturecell Co., Ltd., Seoul 07238, Korea; jcra@stemcellbio.com (J.C.R.); hellena1710@braincell.co.kr (S.H.K.); eunjjan@stemcellbio.com (E.J.J.)

**Keywords:** sperm cryopreservation, canine, amniotic membrane, stem cells, conditioned medium, proteomics

## Abstract

**Simple Summary:**

Mesenchymal stem cells and their derivatives are used in clinical studies for their anti-apoptotic, anti-oxidant, immunomodulatory, and regenerative properties. Their use in reproductive medicine is increasing as they have been proved to be beneficial for infertility treatment. Mesenchymal stem cells can secrete factors that influence biological processes in target tissues or cells; these factors are either directly secreted by the cells or mediated through their derivatives. Although the amniotic membrane is easy to obtain and is a good source of stem cells, clinical trials using amniotic membrane-derived mesenchymal stem cells are still uncommon, especially in reproductive medicine or artificial reproductive technologies. The objective of the present study was to demonstrate the effects of conditioned medium prepared from amniotic membrane-derived stem cells on dog sperm cryopreservation. Our results showed that 10% of the conditioned medium enhanced the quality-related parameters of frozen–thawed sperm cells because of the presence of antioxidants and growth factors in the medium, which probably protected spermatozoa during the freeze–thaw process. These results suggest that conditioned media prepared from amniotic membrane-derived mesenchymal stem cells might have clinical applications in assisted reproductive technologies.

**Abstract:**

This study investigated the effects of conditioned medium (CM) from canine amniotic membrane-derived MSCs (cAMSCs) on dog sperm cryopreservation. For this purpose, flow cytometry analysis was performed to characterize cAMSCs. The CM prepared from cAMSCs was subjected to proteomic analysis for the identification of proteins present in the medium. Sperm samples were treated with freezing medium supplemented with 0%, 5%, 10%, and 15% of the CM, and kinetic parameters were evaluated after 4–6 h of chilling at 4 °C to select the best concentration before proceeding to cryopreservation. Quality-related parameters of frozen–thawed sperm were investigated, including motility; kinetic parameters; viability; integrity of the plasma membrane, chromatin, and acrosome; and mitochondrial activity. The results showed that 10% of the CM significantly enhanced motility, viability, mitochondrial activity, and membrane integrity (*p* < 0.05); however, the analysis of chromatin and acrosome integrity showed no significant differences between the treatment and control groups. Therefore, we concluded that the addition of 10% CM derived from cAMSC in the freezing medium protected dog sperm during the cryopreservation process.

## 1. Introduction

Sperm cryopreservation is used to store sperm samples from cancer patients or endangered species, and for other activities such as breeding, shipping, and research; however, the post-thaw quality obtained is low when compared with the fresh samples [1]. During cryopreservation, cold shock and crystal formation from intra- and extra-cellular water induce cryo-injuries that disturb the sperm plasma membrane integrity and lipid composition, resulting in the leakage of intracellular contents. Consequently, sperm metabolism is reduced [2] and apoptotic-like changes occur in the cells [3,4,5]. In particular, sperm cells are more sensitive to environmental changes because of their limited protein and lipid biosynthetic abilities [6] and the absence of DNA repair mechanism [7]. These events ultimately result in a weakened oxidative stress defense that exposes the sperm cells to reactive oxygen species (ROS). An increase in the ROS production during cryopreservation destroys sperm lipid matrix structures and, subsequently, causes the loss of membrane integrity, an increase in lipid peroxidation, and excessive DNA fragmentation [7,8].

To counteract the consequences of cryo-injuries, chemicals with protective properties can be added to the cells prior to or during cryopreservation. Cyto-protective agents such as cryo-protectants, anti-oxidants, or anti-apoptotic factors, act on different levels to protect cells from cryo-injuries. Anti-apoptotic factors such as the anti-cell death fibroblast-growth-factor inducible kinase (FNK) protein or curcumin prevent cell death [9,10]; cryo-protectants such as glycerol protect cells from intracellular ice formation and reduce osmotic damage [11], while anti-oxidants such as vitamin E protect the sperm ultra-structure, function, and mitochondrial DNA from oxidative stress [12,13]. Therefore, efficient and effective cryopreservation requires the addition of protective chemicals in the freezing media to increase cellular defenses and reduce ROS generation [10,14,15]. However, the commercial and homemade available freezing medium does not fully satisfy the requirements needed for the complete protection of sperm during the cryopreservation process [16]. Although Tris-egg yolk buffer is the most commonly used diluent for mammalian sperm, many studies show that the supplementation of anti-apoptotic factors, anti-oxidants, post-thaw enhancing chemicals, or novel cryo-protective agents is required to get good post-thaw results [1,5,17,18]. Thus, various molecules such as metformin [19], cholesterol [20], or α-tocopherol [21] have been successfully used in canine sperm cryopreservation to reduce oxidative stress, DNA damage [19,20], improve motility [21], and protect plasma membrane and acrosome integrity [20].

Mesenchymal stem cells (MSCs) and their derivatives are commonly used in regenerative medicine and have proved their clinical efficacy in the treatment of infertility [22,23,24]. The MSCs enhance anti-oxidant defenses in several tissues, including testis [24], through the secretion of proteins that reduce ROS production by scavenging free radicals [24,25] and enhance mitochondrial function through the Akt1 pathway [26]. They also secrete anti-inflammatory molecules and growth factors that protect cells from apoptosis when exposed to injuries [27,28]. Amniotic membrane-derived MSCs (AMSCs) have been isolated in dogs and humans [29,30]; although human AMSCs already proved to be useful in regenerative medicine [31], the use of canine AMSCs in this field have only been suggested but never applied [32,33]. In comparison with other stem cells, AMSCs are easier to obtain and isolate, which make them an ideal candidate for clinical trials [31,34].

The effects of MSCs on live tissues are mostly due to their paracrine signaling [35], since their secretome is rich in anti-oxidants and anti-apoptotic factors, which makes them a good alternative to cell therapy [36,37]. The derivatives of MSCs confer the same effects as the cells from which they originate [37], and they have regenerative and protective properties [38] that could positively affect sperm cells. In particular, conditioned medium (CM) has been studied and used in several clinical trials since it is easy to get, safer than cell-based therapies [39], and has a low immunogenicity [39], anti-oxidant and anti-apoptotic properties [40,41,42]. Moreover, CM can be used in many types of research studies since it can be manipulated more easily in comparison with cells [43] and it can also be added in solutions [44].

It is known that MSCs-derived CM obtained from starved cells consists of paracrine factors that enhance cell defense and trigger anti-apoptotic and anti-oxidative mechanisms [45]. The use of these factors may protect sperm from the detrimental effects of cryopreservation such as oxidative stress, apoptosis, DNA damage, and loss of mitochondrial activity. Therefore, we hypothesized that CM prepared from canine amniotic membrane-derived MSCs (cAMSC-CM) would have cyto-protective effects on dog sperm during the freeze–thaw process.

## 2. Materials and Methods

### 2.1. Experimental Design

Experiment 1 focused on the characterization of cAMSCs by flow cytometric analysis and pluripotency genes confirmation, the preparation of cAMSC-CM, and analysis of its components. Experiment 2 was conducted using cAMSC-CM. First, high and low ranges of cAMSC-CM concentrations were added to a freezing medium and used on dog sperm during chilling and cryopreservation process; however, high concentrations of cAMSC-CM had deleterious effects on sperm cells, and therefore, the experiments were conducted using a lower range of concentrations from 0 to 15% of cAMSC-CM. The optimal concentration of cAMSC-CM was selected by evaluating sperm kinetic parameters and viability after 4 to 6 h of chilling in the freezing medium supplemented with cAMSC-CM. Afterwards, in experiment 3, the optimal concentration of cAMSC-CM determined in Experiment 2 was used for dog sperm cryopreservation, and post-thaw quality-related parameters were evaluated and compared with the control group.

### 2.2. Cell Culture

Canine amniotic membrane-derived mesenchymal stem cells (cAMSC) and their culture medium were provided by Naturecell Co., Ltd. (Seoul, Korea). In brief, cAMSCs were seeded and cultured in tissue culture dishes with RCMEP media (Stem Cell Research Center, Biostar, Seoul, Korea), supplemented with serum and antibiotics. Cells were incubated in a humidified environment containing 5% CO_2_ at 37 °C. The cells used for the characterization of cAMSC were cultured until passage two at 90% confluency, and the cells used to make the (CM) were cultured until passage three. All chemicals, unless otherwise stated, were purchased from Sigma-Aldrich (St. Louis, MO, USA).

### 2.3. Flow Cytometric Analysis

Fluorescence-activated cell sorting (FACS) was used to determine cAMSC immunophenotype. Cells were washed two times with phosphate-buffered saline (PBS; Thermo Fisher Scientific, Waltham, MA, USA) TrypLE™ Express (Gibco, Grand Island, NY, USA) was used to detach the cells. Cells were washed with PBS (Thermo Fisher Scientific) two times, counted, and aliquoted in a 96-well plate (1 × 10^5^ cells/100 µL per well). In each well, 5 µL of fluorochrome-conjugated antibodies or isotype control antibodies with fluorescein isothiocyanate (FITC) or phycoerythrin (PE)—CD29 Monoclonal Antibody-PE (Invitrogen, CA, Carlsbad, USA), CD44 Monoclonal Antibody-FITC, CD90 (Thy-1) Monoclonal Antibody-PE, CD34 Monoclonal Antibody-PE, CD45 Monoclonal Antibody-FITC, Rat IgG2a kappa Isotype Control-FITC, Rat IgG2b kappa Isotype Control-PE, Mouse IgG1 kappa Isotype Control-PE, and Rat IgG2b kappa Isotype Control-FITC (eBioscience, CA, San Diego, USA)—were added to the aliquoted cell suspensions. After 30 min of incubation at 4 °C, the cells were centrifuged (1500 rpm/3 min) and washed with PBS two times. Cells were transferred in round tubes with 5 mL of PBS, analyzed using FACSCalibur™, and Cell Quest software (BD Biosciences, CA, San Jose, USA) was used to calculate CD (Cluster of Differentiation) marker percentages. For each antibody, 10,000 cells were used.

### 2.4. Quantitative Polymerase Chain Reaction

Pluripotent genes expression was confirmed by quantitative polymerase chain reaction (qPCR). To summarize, RNA was extracted from cAMSC cultures at passage two using the RNeasy Mini kit (Qiagen, Hilden, Germany). Next, cDNA was synthesized by RNA reverse transcription using DiaStar 2X RT Pre-Mix (Solgent, Daejeon, Korea) and Random Hexamers (Invitrogen, Carlsbad, CA, USA). Synthesized cDNA was amplified by PCR, and real-time PCR was performed using Agilent ariaMX Real-Time PCR (Agilent, Santa Clara, CA, USA). Primers used for qPCR are displayed in Supplementary Materials Table S1 and beta-actin was used as an endogenous control. For gel electrophoresis, 10 µL of each real-time PCR product were loaded in wells and subjected to 1% agarose gel electrophoresis for 20 min.

### 2.5. Conditioned Medium Preparation

The cAMSCs were maintained in their culture media until they reached 80% confluency at passage three. The cells were starved by changing the media to serum-free Dulbecco’s Modified Eagle Medium. After 48 h [46], CM was retrieved, centrifuged (2000× *g*/30 min), and filtered with a 0.22 µm filter to remove cell debris. The CM aliquots were stored at −80 °C.

### 2.6. Proteomic Analysis and CM Composition

To identify and quantify cAMSC-CM protein composition, a one-dimensional electrophoresis-liquid chromatography tandem mass spectrometry (1-DE-LC-MS/MS) system coupled with a Q Exactive Plus mass spectrometer (Thermo Scientific, Waltham, MA, USA) was used. In brief, cAMSC-CM was collected and submitted to precipitation (ppt) for protein purification using ammonium sulfate (AS) saturated at 80%. Then, the CM was centrifuged at 18,000 rpm/1 h for precipitation, dissolved using 20 mM tris-HCl pH 8.0, and AS was removed using Viva spin (50kD). Protein lysates were separated using 12% sodium dodecyl–sulfate polyacrylamide gel electrophoresis (12% SDS-PAGE) followed by in-gel digestion with trypsin. MASCOT software (Matrix Science Inc., MA, Boston, USA) was used to identify the proteins, generate the exponentially modified protein abundance index (emPAI), and then, mole percentage was calculated according to emPAI values. Obtained data were analyzed using the UniProtKB (UniProt Knowledgebase) database (Canis_lupus familiaris). Identified proteins were classified by groups based on their functions, and the mole percentages for each group of proteins present in the cAMSC-CM was calculated.

### 2.7. Animal Use for Semen Collection

All dogs used for the study were kept in individual cages. They were fed with commercial adult dry food, and water was provided ad libitum. All the experiments and studies were conducted in accordance with the recommendations described in “The Guide for the Care and Use of Laboratory Animals” published by the Institutional Animal Care and Use Committee (IACUC) of Seoul National University (approval numbers; SNU-180731-2-1 and SNU-200409-3). Semen was obtained from beagle dogs by using digital manipulation twice a week, and only the second fraction of the ejaculate was collected and processed. The samples with an appropriate concentration (>300 × 10^6^ sperm/mL), motility higher than 80%, and mass motility of 4/5 at least (on a scale of 0–5) were selected and pooled to avoid individual variations. One ejaculate per male was obtained from five males, and four independent replicates were performed using the pooled semen.

### 2.8. Determination of CM Optimal Concentration

The pooled semen was diluted with Tris-extender 1:1 (*v*/*v*)—distilled water, tris (hydroxymethyl) aminomethane 24 g/L, citric acid 14 g/L, fructose 8 g/L, kanamycin sulfate 0.15 g/L; pH 6.6, 290 mOsm—and centrifuged at 700× *g* for 1 min. The supernatant was collected and centrifuged (500× *g*/5 min), and only the pellet was re-suspended in Tris-extender to achieve a concentration of 200 × 10^6^ sperm cells/mL. Different concentrations of CM (0%, 5%, 10%, and 15%) were added to the freezing media—54% (*v*/*v*) Tris-extender, 40% *(v*/*v*) egg yolk, and 6% (*v*/*v*) glycerol —and the samples were chilled in it for 4–6 h at 4 °C. The sperm analysis imaging system (FSA2011 premium edition version 2011; Medical Supply, Gangwon, Korea) was used to evaluate sperm motility, curvilinear velocity (VCL), straight-line velocity (VSL), average path velocity (VAP), linearity (LIN), straightness (or VSL/VAP) (STR), amplitude of lateral head (ALH), and viability parameters. The treated group with the best results was selected for the rest of the experiments, as previously described [47,48].

### 2.9. Semen Cryopreservation and Thawing

The pooled semen was diluted with Tris-extender, washed, and centrifuged. The pellet was re-suspended in Tris-extender to achieve a concentration of 200 × 10^6^ sperm cells/mL. The semen was divided into aliquots to be used for the control and the treatment groups. The freezing medium, with or without CM, was added by a multistep loading protocol [49], and 14%, 19%, 27%, and 40% of the freezing medium was added every 30 s. The samples were loaded in 0.5 mL straws (Minitube, Tiefenbach, Germany) and were kept at 4 °C for 1 h to reach equilibration. Then, they were placed horizontally to freeze at 5 cm above liquid nitrogen (LN_2_) for 15 min, before being transferred to LN_2_ tanks (−196 °C). Then, the semen straws were thawed in a water bath at 37 °C for 30 s. The samples were diluted with Tris-extender (1:5, *v*/*v*) stepwise using 14%, 19%, 27%, and 40% of the total volume at intervals of 30 s. Then, the samples were washed, and afterward, we proceeded with the analysis.

### 2.10. Sperm Kinetic Parameters Analysis

Sperm kinetic parameters were analyzed using a computer-assisted sperm analysis (CASA; Sperm Class Analyzer^®^ System version 6.4.0.93, Microptic, Barcelona, Spain). The system included a Nikon Eclipse ci-L microscope (Nikon, Tokyo, Japan) with a 10× phase-contrast objective and a heating stage at 37 °C. Leja 20 µm chamber slides (Leja, Gynotec Malden, Nieuw Vennep, The Netherlands) were used for the analysis, and the frame rate was set at 25 frames/s. Various parameters such as sperm motility, progressive motility, VCL, VSL, VAP, LIN, STR, ALH, and the percentage of rapid and immotile spermatozoa were analyzed.

### 2.11. Eosin–Nigrosin Staining

Eosin–nigrosin staining was used to determine the percentage of sperm cells alive and tail morphology defects in each group. In brief, the frozen–thawed samples were washed, and a drop of 10 µL from the sperm pellet with an equal amount of eosin and nigrosin was mixed and smeared onto warm glass slides. The slides were then air-dried, and the sperm was evaluated afterward. For each stained smear, 200 sperms were examined with a light microscope (Eclipse Ts 2, Nikon, Tokyo, Japan) with oil immersion objective lens (1000× magnification). The unstained sperms were counted as alive, and the stained ones were counted as dead cells. The results are expressed as the percentage of live sperm cells [47]. Sperm with a coiling of the mid piece were counted as cells with a coiled tail, and sperm with a bending of the mid piece or the entire tail were counted as cells with a bent tail [50].

### 2.12. Aniline Blue Staining

The frozen–thawed samples were washed, and 20 µL of sperm pellet was smeared on a glass slide, air-dried, and fixed with a solution of 3% buffered glutaraldehyde in 0.2 M phosphate buffer (pH 7.2) for 30 min. Then, the slides were stained with 5% aqueous aniline blue solution mixed with 4% acetic acid (pH 3.5) for 5 min. In each group, 200 sperm cells were evaluated with a light microscope (Eclipse Ts 2, Nikon, Tokyo, Japan) in oil immersion objective lens (1000× magnification). The cells with unstained nuclei were considered normal (mature chromatin), and those with blue-stained nuclei were considered abnormal (immature chromatin). The results are expressed as the percentages of aniline blue-positive sperm (abnormal) [51].

### 2.13. Hypo-Osmotic Swelling Test

The hypo-osmotic swelling test (HOST) was performed to evaluate the percentage of sperm cells with an intact plasma membrane. In brief, 100 µL of sperm was added to 900 µL of a hypo-osmotic solution (150–155 mOsm) and incubated at 37 °C for 30 min [52]. Then, a drop of HOST solution with sperm was placed on a warm slide and covered, and at least 100 spermatozoa were counted using a phase-contrast microscope (Eclipse Ts 2, Nikon, Tokyo, Japan). The cells with a coiled tail were counted as HOST-positive sperm.

### 2.14. Acrosome Assessment Test

The sperm acrosome membrane was analyzed using fluorescein isothiocyanate-conjugated peanut agglutinin (FITC-PNA) as described previously. In brief, semen was smeared on glass slides, air-dried, fixed in absolute methanol, stained, and mounted with anti-fade mounting medium (VECTASHIELD^®^, Vector Laboratories, Burlingame, CA, USA). The integrity of sperm acrosome membrane was analyzed using an epifluorescence phase-contrast microscope (Eclipse Ts 2, Nikon, Tokyo, Japan) and classified as intact acrosome (strong green fluorescence) or non-intact acrosome (partial or no fluorescence) [53].

### 2.15. Mitochondria Activity Assessment

The percentage of live sperm cells with functional mitochondria was assessed using a combination of fluorescent stains Rhodamine 123 (R123) (Molecular Probes, OR, Eugene, USA) and propidium iodide (PI) as described previously. In each slide, 200 spermatozoa were examined under an epifluorescence phase-contrast microscope (Eclipse Ts 2, Nikon, Tokyo, Japan) at 600× magnification, equipped with an excitation/barrier filter of 490/515 nm for R123 (blue excitation), excitation/barrier filter of 545/590 nm for PI (green excitation), and a digital camera (Olympus DP 11, Tokyo, Japan). The sperm cells displaying green fluorescence in the mid-piece region and no red fluorescence in the head were considered viable with functional mitochondria, whereas cells exhibiting red fluorescence in the head were counted as dead [54].

### 2.16. Statistical Analysis

Prior to analysis, D’Agostino and Pearson omnibus test was performed. Optimal concentration data were analyzed using one-way analysis of variance (ANOVA) following by a Tukey’s multiple comparison test. For the control and 10% CM-treated groups, the independent sample *t*-test was used. For each experiment, four replicates were performed and the statistical analysis was performed using GraphPad Prism 5.0 (GraphPad, CA, San Diego, USA). The values are expressed as mean ± standard error of the mean (SEM), and the values less than *p* < 0.05 were considered statistically significant.

## 3. Results

### 3.1. cAMSC and cAMSC-CM Characterization

#### 3.1.1. Confirmation of the Surface Markers

**Figure 1:**
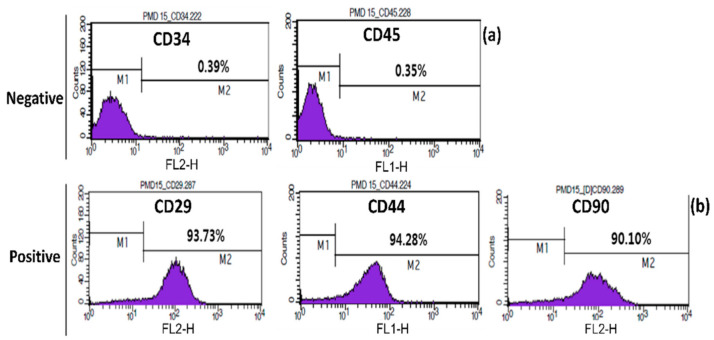
Confirmation of surface markers of canine amniotic membrane-derived mesenchymal stem cells (cAMSC) using fluorescence-activated cell sorting (FACS); (**a**) negative surface markers CD34, CD45; (**b**) cAMSC-positive surface markers CD29, CD44, and CD90 surface makers analyzed by FACS.

Flow cytometry analysis of cAMSC showed that the expression of CD29, CD44, and CD90 markers was high (93.73, 94.28, and 90.10%, respectively), whereas the expression of CD34 and CD45 markers was low (0.39 and 0.35%, respectively) (Figure 1). Therefore, based on the presence of these surface markers, we could infer that these cells were MSCs.

#### 3.1.2. Confirmation of Pluripotency Genes Expression

The qPCR analysis was performed to analyze the expression of pluripotency genes. qPCR results confirmed the expression of pluripotency genes Oct3/4, Sox2, and Nanog in cAMSC (Figure S1), which proves that cAMSCs exhibit pluripotency potential.

#### 3.1.3. cAMSC-CM Proteome

The proteomic analysis showed the presence of 86 proteins (Table S2) and was expressed in mole percentage. Intermediate filaments (26%), other types of proteins involved in cell metabolism (21%), growth factors (18%), extracellular matrix components (15%), anti-oxidants (13%), and enzymes (7%) were found in the cAMSC-CM (Table 1).

### 3.2. Determination of cAMSC-CM Optimal Concentration

Sperm viability and VCL were higher in the treated groups, 5%, 10%, and 15% CM (5% CM, 75.4 ± 6.7% and 83.9 ± 2.8%; 10% CM, 87.2 ± 8.1% and 90.1 ± 2.8%; 15% CM, 75.4 ± 6.7% and 86.8 ± 3.5%, respectively), and the group treated with 10% CM was significantly higher (*p* < 0.05) in comparison with the control group (74.2 ± 4.4% and 80.8 ± 2.0%, respectively). The 10% CM-treated group showed significantly higher motility and ALH (79.2 ± 2.6% and 4.8 ± 0.3 µm) than the other groups (control, 67.3 ± 2.5% and 4.1 ± 0.1 µm; 5% CM, 72.4 ± 2.5% and 4.1 ± 0.1 µm; 15% CM, 72.1 ± 3.9% and 4.1 ± 0.3 µm) (*p* < 0.05), (Table 2). Therefore, the 10% CM-treated group was selected for the rest of the experiment.

### 3.3. cAMSC-CM Effects on Sperm Cryopreservation

#### 3.3.1. Motility and Velocity Parameters

**Figure 2:**
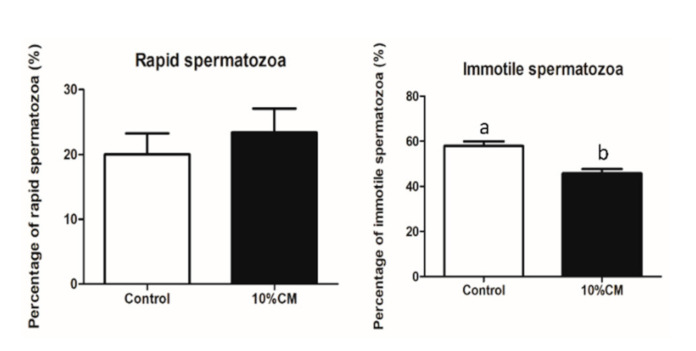
Percentages of rapid and immotile in frozen–thawed sperm. (**a**) Percentage of rapid sperm cells; (**b**) Percentage of immotile sperm cells. Bars with the letters “a” or “b” are values with a statistically significant difference (*p* < 0.05, *n* = 4).

The CASA system results showed that 10% CM treatment significantly enhanced (*p* < 0.05) motility and LIN (54.3 ± 1.9% and 50.3 ± 3.1%, respectively) of sperm compared to that of the control group (42.1 ± 2.1% and 47.0 ± 3.4%, respectively). The percentage of immotile spermatozoa was significantly reduced in the treatment group (45.7 ± 1.9%) when compared with the control group (57.9 ± 2.1%) (*p* < 0.05), (Figure 2). The rest of the parameters showed no significant differences between the groups (*p* < 0.05) *(*Table 3).

#### 3.3.2. Live/ Dead Count and Morphology Assessment

The percentage of live sperm cells was higher in the 10% CM-treated group (55.2 ± 3.0%) than that of the control group (43.9 ± 4.3%), and the percentage of spermatozoa with a bent tail was lower in the treatment group (1.8 ± 0.6%) than the control group (3.1 ± 0.7%) (*p* < 0.05), (Table 3). However, the percentage of cells with a coiled tail was not significantly different between the control and the 10% CM-treated groups (3.0 ± 1.2% and 3.0 ± 1.8%, respectively). These results suggest that cAMSC-CM probably has an anti-apoptotic effect during sperm cryopreservation.

#### 3.3.3. Chromatin Integrity

The percentage of spermatozoa with abnormal chromatin condensation-stained nuclei was not significantly different in both the groups (control, 34.0 ± 2.9%; 10% CM, 31.0 ± 3.1%) (*p* < 0.05), (Table 4). These results suggest that cAMSC-CM has no protective effect on DNA integrity.

#### 3.3.4. Acrosome and Membrane Integrity Assessment

The percentage of sperm cells with intact plasma membrane was significantly higher (*p* < 0.05) in the treatment group (66.5 ± 2.3%) than the control group (54.5 ± 2.9%). The FITC-PNA test revealed no significant difference (*p* < 0.05) in the percentage of spermatozoa with an intact acrosome between the two groups (control, 74.0 ± 4.3%; 10% CM, 76.6 ± 4.0%) (Table 5).

#### 3.3.5. Mitochondria Activity Assessment

**Figure 3:**
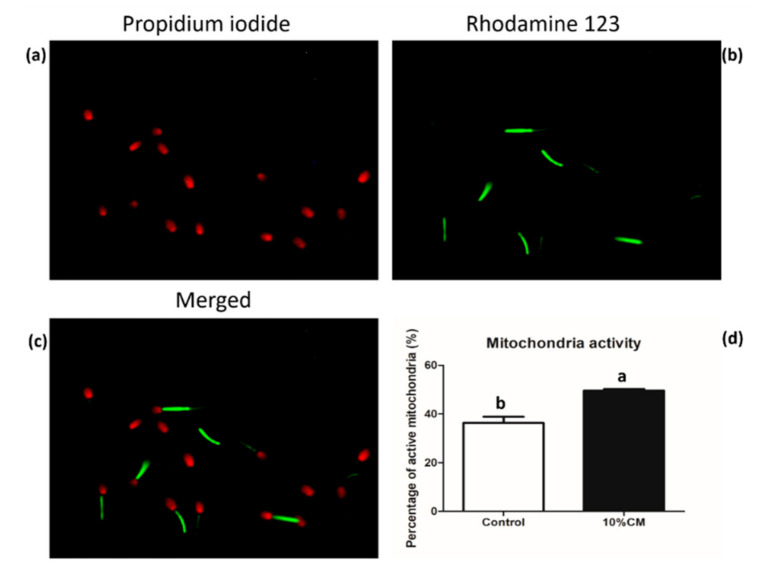
Mitochondria activity in frozen–thawed sperm using rhodamine 123 (R123) and propidium iodide (PI) dual staining; (**a**) dead sperm stained with PI; (**b**) live sperm with R123-stained mitochondria; (**c**) merged channels showing both PI and R123 stained sperm; (**d**) percentage of sperm with active mitochondria. Bars with the letters “a” or “b” are values with a statistically significant difference (*p* < 0.05, *n* = 4).

The R123 dye was used to assess mitochondrial activity, and PI was used to stain dead sperm cells. Both the data were used to calculate the percentage of live sperm with active mitochondria in each group. The 10% CM-treated group showed significantly enhanced (*p* < 0.05) mitochondrial activity (49.6 ± 0.7%) compared with the control group (36.4 ± 2.5%) (Figure 3).

## 4. Discussion

Since the isolation of MSCs from amniotic tissues, researchers have displayed a growing interest in the study of their characteristics, properties, and possible applications [31,34,55]. Thus, they have become an interesting alternative to embryonic stem cells, as they can be obtained by non-invasive methods [31], and the ethical issues associated with the use of amniotic tissues-derived stem cells are minor [34], since they are obtained from the placenta that is usually discarded after caesarian sections [29]. They are non-teratogenic [55], pluripotent, and also have regenerative properties and low immunogenicity [31,34,55]. Canine MSCs derived from the amniotic membrane have recently been isolated and proved to have the same characteristics, biosafety, and properties as other MSCs, which make them a good candidate for clinical trials [32,56]. The composition and potential use of cAMSC-CM have not been studied yet, but since CM contains cell paracrine factors [37], the application of cAMSC-CM in clinical studies seems to be promising. In our study, cAMSC phenotype was confirmed through FACS analysis and showed a high expression of CD29, CD44, and CD90 surface markers, whereas CD34 and CD45 expression, which are associated with hematopoietic stem cells [57,58], was low (Figure 1). This phenotype has also been found in other studies using canine MSCs [59,60,61], and CD29, CD44, and CD90 surface antigens are found on adipose, bone marrow, and umbilical cord blood-derived mesenchymal stem cells [62,63]. Pluripotency is an important factor in MSCs, since it is involved in regenerative pathways [64]. The analysis of pluripotency gene expression (*Oct3/4*, *Nanog*, and *Sox2*) also confirmed the MSC phenotype (Figure S1). The analyzed genes are essential for the self-renewal of MSCs, determination of pluripotency and maintenance of cells’ undifferentiated state [65,66]. Their expression by cAMSCs is an indicator that cAMSCs could be used in regenerative medicine.

Stem cells-derived CM contains growth factors and anti-apoptotic factors, and it has been used in cell-free therapies to mediate stem cells paracrine effects on tissues [39,41,42]. Each CM has a different composition depending on the cells of origin, cell state, and the microenvironment surrounding them [35]. Previously, canine MSC-derived CM was used for different clinical applications and showed good results in xenogeneic tissues wound healing [67], laryngotracheal stenosis healing [68], and stem cells’ survival and differentiation [69]. However, no study has unveiled the effects of cAMSC-CM and its proteomic analysis. In this study, the composition of cAMSC-CM was investigated, and the proteomic analysis revealed the presence of intermediate filaments, extra-cellular matrix components (ECM), anti-oxidants, growth factors, enzymes, and other proteins involved in the cell metabolism (Table S2, Table 1). Our results corroborate those of previous studies showing that two of the main components of CMs were growth factors and anti-oxidants [39]. Furthermore, an amniotic membrane stem cells proteome from another study [70] showed the presence of ECM components involved in cellular processes through the focal adhesion kinase signaling pathway such as lumican, collagen, and fibronectin, which were also found in cAMSC-CM in our study (Table S2, Table 1). Apolipoproteins, especially Apolipoprotein A-1, which is involved in sperm capacitation and motility [71]; and Apolipoprotein E, which has an anti-oxidant activity [72], were also found in cAMSC-CM (Table S2). Some of the growth factors found in our study include thioredoxin, which has an anti-oxidant activity and plays a role in fertility [73,74,75], and serum albumin, which might also play an important role in fertility improvement [76] and sperm cryopreservation, by enhancing post-thaw motility and protecting sperm morphology, and the integrity of the plasma membrane, acrosome, and DNA [77,78].

Although sperm cells have the ability to adapt to osmotic changes [79], freezing medium does not fully protect them from the osmotic stress happening during freezing and thawing that leads to an increase in ROS production, apoptosis-like changes, DNA damage, and an increase in tail defects [80,81,82]. Therefore, we hypothesized that the addition of cAMSC-CM to the freezing medium would increase sperm tolerance to osmotic changes, oxidative defence, and help protect their ultra-structure, because it contains proteins with anti-oxidant, regenerative, and anti-apoptotic effects (Table S2). To date, no study has depicted the addition of CM in sperm cryopreservation, and the range of CM concentrations added to the freezing medium remains unknown. Here, we evaluated the optimal concentration of CM starting from a low range of concentrations, from 0 to 15% of the CM, among which 10% of the CM revealed to be the best concentration (Table 2). We assumed that a concentration higher than 15% would influence sperm homeostasis, as it would probably change the osmolarity of the milieu. In fact, during our preliminary study, we observed that the use of a higher range of concentrations (from 25 to 75%) negatively impacted the quality-related parameters of frozen–thawed sperms (data not shown). Nevertheless, from the low range of concentrations, the supplementation of 10% CM in the freezing medium was found to be the optimal one and was further used for cryopreservation.

Our results showed that the percentage of live sperm was significantly increased when cAMSC-CM was added to the freezing medium (Table 4), which could be explained by the regenerative and anti-apoptotic effects of cAMSC-CM proteins. When CM is prepared, the components released from the starvation phase resulting from the MSC survival state activation causes the release of protective factors that protects cells from apoptosis and oxidative stress [45]. These factors probably improved the anti-oxidant defence and anti-apoptotic mechanisms in sperm. However, they were not sufficient to protect chromatin integrity, as aniline blue test results showed no significant differences between the control and cAMSC-CM treated groups. Aniline blue dye binds to nucleoproteins and allows researchers to evaluate chromatin integrity; however, DNA denaturation and fragmentation induced by freezing and thawing are not always immediately apparent. A study showed that canine sperm further incubated after thawing showed an increase in the DNA fragmentation index [83].

The cryopreservation process leads to an increase in sperm tail abnormalities [80,84], altered acrosome and plasma membrane integrity [85], and a decrease in the mitochondrial activity [4,82] and motility [80,82]. In our study, we found that the percentage of coiled tail sperm was the same in both groups, but the percentage of sperm with a bent tail was significantly reduced in the 10% CM-treated group in comparison with the control group (Table 4). The proteins present in seminal plasma can interact with sperm at the surface and repair plasma membranes and mitochondrial DNA [86]. In our study, some of the proteins found in cAMSC-CM, including collagen, olfactory receptors, zinc finger protein, vitamin D binding protein, fibronectin, and serum albumin (Table S2), have also been found in the seminal plasma collected from fertile men [87], boar [88,89], alpaca, and camels [89]. These proteins, along with the protective nature of other cAMSC-CM factors, might explain the positive effects on sperm ultra-structural characteristics (Table 4). Moreover, the positive correlation between some of these proteins and fertility [76,87] and their role in mitochondrial DNA repair [86] might explain post-thaw improved sperm motility, LIN (Table 3), and the enhanced mitochondrial activity (Figure 3). Mitochondrial activity is essential for sperm motility, and a decrease in its activity results in increased apoptosis [90]. Furthermore, the addition of 10% cAMSC-CM reduced the percentage of immotile sperm (45.7 ± 1.9%) (Figure 2). We can hypothesize that proteins in cAMSC-CM might have reduced ROS production and have had a positive role in mitochondrial DNA repair. However, the percentage of sperm with an intact acrosome was high in both the groups (74.0 ± 4.3% and 76.6 ± 4.0% respectively), but there was no significant difference between them (Table 6). A study showed that acrosome integrity is above 60% in canine sperm at 0 h after thawing, when 6–8% glycerol is used [91]. In our freezing medium, 6% glycerol was used, and the acrosome integrity test was performed within few minutes after thawing. This might explain the high percentage of intact acrosomes observed in both groups.

The preservation of sperm membrane integrity and mitochondrial function during the freeze–thaw process is important for successful fertilization, but the commercial freezing media do not protect sperm from the loss of these functions [16]. The addition of cyto-protective factors in the freezing medium can prevent the deleterious effects of cryopreservation [92], because they can protect cells from the negative effects of ROS on sperm motility, mitochondrial activity, and DNA integrity [93]. In our study, the preservation of membrane integrity was more effective in the 10% CM-treated group (66.5 ± 2.3%) in comparison with the control group (54.5 ± 2.9%). This might be due to the proteins present in cAMSC-CM, especially apolipoproteins (Table S2). ROS targets polyunsaturated fatty acids and cholesterol [94], which disturbs the integrity of the plasma membrane that is essential to sperm homeostasis [95]. In particular, cholesterol is important for membrane stability and can determine sperm freezability, as a disturbed cholesterol to phospholipid ratio can negatively impact the outcome of cryopreservation [96]. Apolipoproteins play a crucial role in cholesterol homeostasis in the epididymis [97], and they are involved in lipid exchange, capacitation, and membrane remodeling [71,98]. In addition, fibronectin, an ECM component that acts as a growth factor [99], was found in the cAMSC-CM, which is also involved in protecting membrane integrity (Table S2 and Table 1). Qamar et al. [100] showed that fibronectin was expressed in adipose-derived MSCs and that plasma membrane integrity was protected when adipose-derived MSCs were added to the freezing media. This suggests that cAMSC-CM protected plasma membrane integrity in the present study (Table 6).

## 5. Conclusions

In conclusion, our results showed that cAMSC-CM contained proteins, including growth factors, anti-oxidants, enzymes, and ECM components, which protect sperm functions and ultra-structure characteristics during cryopreservation. The addition of cAMSC-CM in the freezing medium enhanced sperm motility and viability, membrane integrity, and mitochondrial activity. This was the first study to reveal the composition of cAMSC-CM and its effect on sperm cryopreservation. Further in vitro studies to evaluate cAMSC-CM effects on sperm capacitation and fertilizing ability should be conducted. In vivo studies should also be conducted to reveal the effects of cAMSC-CM proteins on fertility, along with a much deeper study on the proteins implicated in canine sperm fertility.

## Figures and Tables

**Table 1:** Mole percentages of canine amniotic membrane-derived mesenchymal stem cells CM components from proteomics analysis.

Type of Proteins	Total Mole Percentage by Type of Proteins (%)
Intermediate filaments	26
Cell metabolism	21
Growth factors	18
Extra-cellular matrix components	15
Anti-oxidants	13
Enzymes	7

**Table 2:** Motility and velocity parameters of 4 h chilled sperm using different concentrations of canine amniotic membrane-derived mesenchymal stem cells CM.

Concentration of CM (%)	Motility (%)	Viability (%)	^1^ VCL (µm/s)	VSL (µm/s)	VAP (µm/s)	LIN (%)	STR (%)	ALH (µm)
0	67.3 ± 2.5 ^b^	80.8 ± 2.0 ^b^	74.2 ± 4.4 ^b^	21.4 ± 1.3	45.8 ± 2.0	29.0 ± 1.3	47.4 ± 1.7	4.1 ± 0.1 ^b^
5	72.4 ± 2.5 ^b^	83.9 ± 2.8 ^ab^	75.4 ± 6.7 ^ab^	20.4 ± 1.0	46.8 ± 3.1	29.0 ± 1.5	44.1 ± 1.4	4.2 ± 0.3 ^b^
10	79.2 ± 2.6 ^a^	90.1 ± 2.8 ^a^	87.2 ± 8.1 ^a^	23.8 ± 1.8	54.0 ± 4.0	31.2 ± 1.6	44.5 ± 1.2	4.8 ± 0.3 ^a^
15	72.1 ± 3.9 ^b^	86.8 ± 3.5 ^ab^	75.4 ± 6.7 ^ab^	24.6 ± 3.7	46.7 ± 4.0	31.6 ± 2.1	46.4 ± 2.3	4.1 ± 0.3 ^b^

^1^ VCL, average curvilinear velocity; VSL, straight-line velocity; VAP, average path velocity; LIN, linearity (average ratio of VSL/VCL); STR, straightness (average value of the ratio VSL/VAP); ALH, amplitude of lateral head. All results show means ± SEM. Values within marked with the letters “a” or “b” are significantly different (*p* < 0.05, *n* = 4).

**Table 3:** Motility and velocity parameters of frozen–thawed sperm in control and 10% of canine amniotic membrane-derived mesenchymal stem cells conditioned media (CM) treatment groups.

Concentration of CM (%)	Motility (%)	Progressive Motility (%)	^1^ VCL (µm/s)	VSL (µm/s)	VAP (µm/s)	LIN (%)	STR (%)	ALH (µm)
0	42.1 ± 2.1 ^b^	22.8 ± 3.4	81.5 ± 6.4	49.4 ± 5.6	57.2 ± 5.6	47.0 ± 3.4 ^b^	68.1 ± 2.4	3.1 ± 0.3
10	54.3 ± 1.9 ^a^	26.2 ± 4.2	74.5 ± 7.8	46.3 ± 7.1	53.3 ± 7.2	50.3 ± 3.1 ^a^	70.0 ± 2.2	2.8 ± 0.2

^1^ VCL, average curvilinear velocity; VSL, straight-line velocity; VAP, average path velocity; LIN, linearity (average ratio of VSL/VCL); STR, straightness (average value of the ratio VSL/VAP); ALH, amplitude of lateral head. Values are presented as means ± standard error of the mean (SEM). Values within columns marked with the letters “a” or “b” are significantly different (*p* < 0.05, *n* = 4).

**Table 4:** Live sperm percentage and morphological defects of frozen–thawed sperm in control and 10% of canine amniotic membrane-derived mesenchymal stem cells CM treatment groups.

Concentration of CM (%)	Live Sperm Cells (%)	Coiled Tail (%)	Bent Tail (%)
0	43.9 ± 4.3 ^a^	3.0 ± 1.2	3.1 ± 0.7 ^a^
10	55.2 ± 3.0 ^b^	3.0 ± 1.8	1.8 ± 0.6 ^b^

Values are presented as means ± standard error of the mean (SEM). Values within columns marked with the letters “a” or “b” are significantly different (*p* < 0.05, *n* = 4).

**Table 5:** Percentage of frozen–thawed sperm with an abnormal chromatin condensation in control and 10% of canine amniotic membrane-derived mesenchymal stem cells conditioned media (CM) treatment groups.

Concentration of CM (%).	Aniline Blue Positive Spermatozoa (%)
0	34.0 ± 2.9
10	31.0 ± 3.1

All results show means ± SEM (*n* = 4).

**Table 6:** Percentages of intact acrosome and membrane in frozen–thawed sperm in control and 10% of canine amniotic membrane-derived mesenchymal stem cells CM treatment groups.

Concentration of CM (%)	Intact Acrosome (%)	Intact Membrane (%)
0	74.0 ± 4.3	54.5 ± 2.9 ^b^
10	76.6 ± 4.0	66.5 ± 2.3 ^a^

Values are presented as means ± standard error of the mean (SEM). Values within columns marked with the letters “a” or “b” are significantly different (*p* < 0.05, *n* = 4).

## Supplementary Materials

The following are available online at https://www.mdpi.com/2076-2615/10/10/1899/s1, Figure S1: Confirmation of pluripotent genes expressions in all cAMSC cell lines (*n* = 11) using quantitative polymerase chain reaction (qPCR); Table S1. List of primers used for quantitative polymerase chain reaction (qPCR); Table S2. Proteins found in canine amniotic membrane-derived mesenchymal stem cells (AMSC) derived conditioned medium.
