# Supplementary material for: Conditioned Medium from Canine Amniotic Membrane-Derived Mesenchymal Stem Cells Improved Dog Sperm Post-Thaw Quality-Related Parameters
Source: Animals (Basel). 2020 Oct 16;10(10):1899. doi: 10.3390/ani10101899 (PMC7603003; doi:10.3390/ani10101899)
Supplement: Supplementary file 1 [file animals-10-01899-s001.pdf]

animals

MDPI

Article

Conditioned Medium from Canine Amniotic Membrane-Derived Mesenchymal Stem Cells Improved Dog Sperm Post-Thaw Quality-Related Parameters

Feriel Yasmine Mahiddine [1], Jin Wook Kim [1], Ahmad Yar Qamar [2,3,4], Jeong Chan Ra [5], Soo Hyun Kim [5], Eun Joong Jung [5] and Min Jung Kim [1,*]

1 Department of Theriogenology and Biotechnologies, College of Veterinary Medicine, Seoul National University, Seoul 08826, Korea; yasmini19@snu.ac.kr (F.Y.M.); vet_chris@snu.ac.kr (J.W.K.)
2 Laboratory of Theriogenology, College of Veterinary Medicine, Chungnam National University, Daejeon 34134, Korea; ahmad.qamar@uvas.edu.pk
3 Department of Clinical Sciences, College of Veterinary and Animal Sciences, Jhang 35200, Pakistan
4 Department of Clinical Sciences, Sub-Campus University of Veterinary and Animal Sciences, Lahore 54000, Pakistan
5 Cell Physiology Research Center, Naturecell Co., Ltd., Seoul 07238, Korea; jcra@stemcellbio.com (J.C.R.); hellena1710@braincell.co.kr (S.H.K.); eunjjan@stemcellbio.com (E.J.J.)
* Correspondence: tinia19@snu.ac.kr; Tel.: +82-2-880-1180

Received: 08 September 2020; Accepted: 14 October 2020; Published: date

Supplemetary Data

Confirmation of pluripotency genes expression

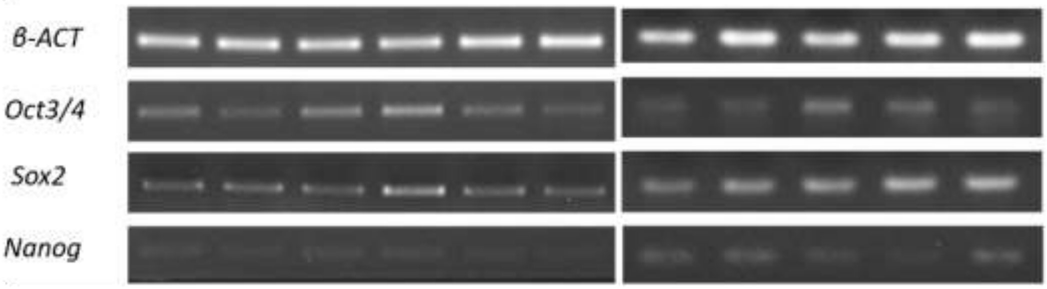

Figure S1. Confirmation of pluripotent genes expressions in all canine amniotic membrane-derived mesenchymal stem cell lines (cAMSC) (n = 11) using qPCR.

Table S1. List of primers used for quantitative polymerase chain reaction (qPCR).

Gene	Primer Sequence (5′-3′)	Product size (bp)	Temperature (°C)	NCBI Accession No.
β-ACT [1]	F - GCTACGTCGCCCTGGACTTC<br>R - GCCCGTCGGGTAGTTCGTAG	86	60	AF021873.2
Oct3/4 [2]	F - CGAGTGAGAGGCAACCTGGAGA<br>R - CCACACTCGGACCACATCCTTC	114	60	XM_538830
Sox2 [3]	F - CAGACCTACATGAACGGCTCGC<br>R - CCTGGAGTGGGAGGAGGAGGTA	147	60	XM_005639752
Nanog [4]	F - GAATAACCCGAATTGGAGCA<br>R - AGTTGTGGAGCGGATTGTTC	125	60	XM_022411387

[1] β-*ACT*, beta-actin; [2] *Oct3/4*, octamer binding transcription factor 3/4, [3] *Sox2*, SRY (sex determining region Y)-box 2, [4] *Nanog*, homeobox protein.

animals
MDPI

Table S2. Proteins found in canine amniotic membrane-derived mesenchymal stem cells (AMSC) derived conditioned medium.

Category / Function	Protein name	Gene	Molecular weight (Da)	Accessions
Extracellular matrix component, growth factor, anti-apoptosis, calcium ion binding	Secreted protein acidic and cysteine rich/Osteonectin	SPARC	35390	E2RMA3
Growth factor, anti-oxidant, metal binding	Serum albumin	ALB	70558	F2Z4Q6
Intermediate filament	Keratin, type I cytoskeletal 10	KRT10	57821	F1PYU9
Extra-cellular matrix component, cell proliferation	Lumican	LUM	38665	E2R416
Intermediate filament	Keratin, type II cytoskeletal 1	KRT1	63846	F1PTY1
Growth factor, glycoprotein, steroidogenesis stimulation	Metalloproteinase inhibitor 1	TIMP1	29250	F1PQS2
Growth factor	Insulin like growth factor binding protein 7	IGFBP7	29907	E2RNL2
Apolipoprotein, cholesterol metabolism, anti-oxidant	Apolipoprotein E	APOE	37258	F1PJ74
Intermediate filament	Keratin 14	KRT14	52621	F1Q0R0
Cell metabolism	Actin, cytoplasmic 1	ACTB	O18840	42052
Intermediate filament	Vimentin	VIM	53622	F1PLS4
Intermediate filament	Keratin 75	KRT75	59713	E2R917
Extra-cellular matrix component	Follistatin like 1	FSTL1	36073	F1PY69
Intermediate filament	Uncharacterized protein	KRT5	62919	E2R8Z5
Extra-cellular matrix component	Collagen type III alpha 1 chain	COL3A1	139948	F1PG69
Intermediate filament	Uncharacterized protein	KRT6A	60975	F1PTS8
Anti-oxidant	Thioredoxin	TXN	11665	J9NWJ5
Intermediate filament	Uncharacterized protein	KRT42	50182	E2R7W6
Enzyme	Malate dehydrogenase	MDH1	38564	E2QV08
Growth factor	Alpha 2-HS glycoprotein	AHSG	40021	E2QUV3
Cell metabolism	GC, vitamin D binding protein	GC	55997	F1P841
Cell metabolism	Plasminogen activator, urokinase receptor	PLAUR	27149	E2RGN0
Cell metabolism	Alpha-fetoprotein	AFP	70491	F1PXN2
Cell metabolism	Uncharacterized protein	YWHAZ	27899	F1PBL1
Apolipoprotein, sperm motility, cholesterol metabolism	Apolipoprotein A-I	APOA1	30178	P02648
Intermediate filament	Uncharacterized protein	KRT4	64360	E2QUU4
Intermediate filament	Keratin, type I cytoskeletal 9	KRT9	65274	F1Q0N7
Cell metabolism	Uncharacterized protein	LOC477072	112612	F6V1W9
Enzyme	Triosephosphate isomerase	TPI1	32085	A0A0A0MPD0
Enzyme	Purine nucleoside phosphorylase	PNP	32566	F1PQM1
Cell metabolism	Hemoglobin subunit beta	HBB	16100	P60524
Growth factor	Fibronectin	FN1	270608	F1P6H7
Extra-cellular matrix component	Collagen alpha-1(I) chain	COL1A1	140042	F1Q3I5
Intermediate filament	Keratin 24	KRT24	56819	E2R150
Enzyme	ATP synthase subunit d, mitochondrial	ATP5PD	18679	J9P8U0

Anti-oxidant	Ferritin	LOC102154317	19901	E2RRH1
Anti-oxidant	Decorin	DCN	40297	Q29393
Extra-cellular matrix component	Uncharacterized protein	LGALS3BP	63197	E2RKQ6
Intermediate filament	Keratin, type II cytoskeletal 2 epidermal	KRT2	65016	F1PTX4
Cell metabolism	SAP domain containing ribonucleoprotein	SARNP	23759	F1PVE3
Extra-cellular matrix component	Dermatopontin	DPT	24578	E2RPQ6
Cell metabolism	Proteasome subunit beta	PSMB6	25686	E2R0B6
Intermediate filament	Keratin 78	KRT78	56429	E2R4B0
Cell metabolism	Uncharacterized protein	LOC475521	26945	F1PCE8
Intermediate filament	Keratin 72	KRT72	57646	E2QUT7
Enzyme	Nicotinate-nucleotide pyrophosphorylase [carboxylating]	QPRT	31301	J9NZ55
Cell metabolism	Growth arrest specific 2	GAS2	35495	F1PBV2
Cell metabolism	Olfactory receptor	OR6K6	38505	E2R0T9
Cell metabolism	Olfactory receptor	LOC100687010	35594	F1PU69
Cell metabolism	Lysosomal protein transmembrane 4 beta	LAPTM4B	37360	F1PGH9
Growth factor	Fetuin B	FETUB	43162	E2R9B6
Enzyme	L-lactate dehydrogenase	LDHA	40058	F1PVW0
Enzyme	L-lactate dehydrogenase	LDHB	36931	J9NT18
Cell metabolism	Tropomyosin 1	TPM1	37436	F1P912
Cell metabolism	Annexin A2	ANXA2	38915	Q6TEQ7
Extra-cellular matrix component	Collagen alpha-2(I) chain	COL1A2	129835	O46392
Growth factor	Cellular communication network factor 2	CCN2	39909	J9NTX8
Extra-cellular matrix component	Thrombospondin 1	THBS1	133521	F1PBI6
Extra-cellular matrix component	Biglycan	BGN	42268	G1K2D8
Cell metabolism	Serpin family A member 7	SERPINA7	46789	F1PB85
Cell metabolism	Serpin family F member 1	SERPINF1	46586	F2Z4Q7
Cell metabolism	Nerve growth factor receptor	NGFR	47024	F1PAR7
Cell metabolism	Mitogen-activated protein kinase associated protein 1	MAPKAP1	59657	E2RAY0
Cell metabolism	Serpin family C member 1	SERPINC1	53003	E2RES2
Cell metabolism	Uncharacterized protein	ZNF385C	53432	F1PZ48
Extra-cellular matrix component	Collagen type V alpha 2 chain	COL5A2	145807	F1PG08
Enzyme	Dihydropyrimidinase like 3	DPYSL3	62236	F1Q3Y2
Cell metabolism	Fragile X mental retardation 1	FMR1	69446	F1Q2U8
Enzyme	TBK1 binding protein 1	TBKBP1	67443	F1P609
Enzyme	Protein phosphatase 1 regulatory subunit	PPP1R12C	85052	E2QUK2
Anti-oxidant	Uncharacterized protein	A2M	166540	F6UME0
Cell metabolism	Potassium calcium-activated channel subfamily N member 2	KCNN2	93690	F1PA33
Cell metabolism	Otoancorin	OTOA	127728	E2RIN3

Cell metabolism	KIAA0319	KIAA0319L	106022	F6XGU5
Other, binding protein	Latent transforming growth factor beta binding protein 1	LTBP1	178675	F1PLA1
Cell metabolism	Rho guanine nucleotide exchange factor 40	ARHGEF40	175931	F1PME6
Enzyme, energy metabolism	Adenylate cyclase 10	ADCY10	189494	E2RDZ3
Extra-cellular matrix component	Collagen type V alpha 1 chain	COL5A1	180707	F1PHX8
Cell metabolism	Fibronectin type III domain containing 3B	FNDC3B	134875	E2REV4
Cell metabolism	Uveal autoantigen with coiled-coil domains and ankyrin repeats	UACA	163865	Q9GL21
Growth factor	Fms related tyrosine kinase 1	FLT1	152451	F1PKV5
Cell metabolism	Zinc finger protein 142	ZNF142	211301	J9P6V2
Cell metabolism	Ryanodine receptor 1	RYR1	572146	F1PIS0
Enzyme	Citron rho-interacting serine/threonine kinase	CIT	236845	E2RPN3
Extra-cellular matrix component	Laminin subunit alpha 3	LAMA3	376682	E2RPP1
Enzyme	DNA-dependent protein kinase catalytic subunit	PRKDC	476572	F1Q3H1
